# Changes in Cerebellar Multiunit Activity Associated with Ventrolateral Striatal Injury During Spontaneous Motor Behavior

**DOI:** 10.3390/medsci14010083

**Published:** 2026-02-11

**Authors:** Irais Viveros-Martínez, Cristofer Zarate-Calderon, Lizbeth Vásquez Celaya, Consuelo Morgado-Valle, María Leonor López-Meraz, Donají Chi-Castañeda, Luis I. García

**Affiliations:** Instituto de Investigaciones Cerebrales, Universidad Veracruzana, Av. Dr. Luis Castelazo Ayala, Col. Industrial Las Ánimas, Xalapa 91190, Veracruz, Mexico

**Keywords:** cerebellum, Crus II, dentate nucleus, inferior olive, Multi-Unit Activity, Parkinsonism, spontaneous behavior, ventrolateral striatum

## Abstract

**Background:** Parkinsonism entails pronounced basal ganglia dysfunction, but emerging research suggests that broader subcortical networks, specifically the cerebellum, play a vital role in functional motor compensation following circuit-level destabilization. This study sought to characterize the electrophysiological dynamics of Multi-Unit Activity (MUA) amplitude in Crus II, the dentate nucleus (DN), and the inferior olive (IO) following a focal mechanical lesion of the ventrolateral striatum (VLS) as a circuit-level perturbation model during spontaneous behaviors. **Methods:** Bilateral mechanical VLS lesions were induced in 24 male Wistar rats. MUA signals were chronically recorded over a four-week protocol during self-grooming, horizontal locomotion, and rearing behaviors. **Results:** Crus II and the IO exhibited a structure-specific “dynamic transition,” shifting from early-stage hyperexcitability to significant late-stage attenuation by W4 (*p* < 0.001), reflecting a divergence from control trajectories rather than internal temporal drift within the lesioned state. Conversely, the DN showed sustained hypoactivity compared to healthy controls throughout the recording period (*p* < 0.05). Despite these robust neurophysiological shifts, the syntactic organization of grooming and exploratory patterns remained phenotypically preserved, indicating functional sufficiency despite underlying circuit noise. **Conclusions:** VLS injury triggers a rapid distributed reorganization across the striato-cerebellar network. The cerebellum acts as an active adaptive node, recalibrating internal network gain to mask early Parkinsonian-like circuit dysfunction at the level of functional sufficiency and maintain motor performance through active homeostatic gain regulation.

## 1. Introduction

Parkinsonism represents a sophisticated clinical spectrum where the quintessential motor feature is bradykinesia, usually presenting alongside cardinal signs such as muscular rigidity, resting tremors, gait disturbances, and postural instability [1,2,3]. These motor manifestations are consistently associated with pronounced dysfunction within the basal ganglia circuits, highly specialized structures essential for the selection, execution, and organization of coordinated movement [3,4]. Beyond these hallmark symptoms, Parkinsonism is characterized by significant alterations in the fluidity and sequencing of spontaneous motor behaviors, suggesting the active involvement of more extensive subcortical motor networks extending beyond the traditional nigrostriatal axis [3,4,5].

In animal models, tremulous jaw movements (TJM) are a robust, widely validated paradigm for examining Parkinsonian-like tremors. These movements have provided a robust platform for investigating functional alterations in motor circuits under conditions of striatal dysfunction [6,7]. Consequently, this experimental framework enables high-resolution analysis of changes in neural activity across subcortical and cerebellar structures, providing an ideal setting for electrophysiological studies of the functional reorganization of motor circuits.

Spatial exploration represents a pivotal spontaneous behavior through which rodents acquire essential information about their environment. This behavior is closely linked to spatial memory processes and the orientation of directional systems, requiring precise integration among motor control, posture, and multi-dimensional coordination [8]. Similarly, self-grooming is an innate behavior involved in functions such as hygiene and thermoregulation and also constitutes a robust model of complex, sequential motor behavior. In rodents, grooming is organized into syntactic chains of stereotyped movements whose precise execution depends on the integrity of the striatum [8]. Indeed, alterations in this structure affect the temporal organization of the behavior without necessarily modifying its overall frequency [9,10].

Extensive evidence indicates that striatal spiny projection neurons (SPNs) play a central role in specifying both the content and the hierarchical structure of motor behavior. In particular, specific neuronal correlations with kinematic parameters, such as body velocity, orientation, and turning, during spontaneous activity, have been identified, highlighting the importance of the striatum in regulating motor dynamics [11]. In this context, spontaneous behavioral states provide a valuable functional framework to evaluate the activity of motor circuits without the confounding effects of directed experimental demands [11,12].

Concurrently, recent research has emphasized the bidirectional functional connectivity between the cerebellum and the basal ganglia [5]. This evidence suggests that cerebellar structures such as Crus II, the inferior olive (IO), and the dentate nucleus (DN) may contribute significantly to Parkinsonian symptomatology through compensatory and adaptive mechanisms within distal motor circuits [13]. This integrated network-level functional interaction positions these cerebellar regions as critical nodes for understanding the motor reorganization associated with striatal dysfunction.

Based on this conceptual framework, the present study aimed to systematically characterize the electrophysiological responses by recording the amplitude dynamics of Multi-Unit Activity (MUA) in key cerebellar regions (Crus II, DN, and IO) during exploratory horizontal and vertical (locomotion and rearing) and self-grooming behaviors in rats subjected to a focal mechanical lesion of the ventrolateral striatum (VLS). In this context, we prioritized Crus II because the posterolateral cerebellar region receives substantial cerebrocortical and pontine inputs that are crucial for sensorimotor integration and have previously been implicated in adaptive cerebellar responses to basal ganglia dysfunction [3,14].

This methodology facilitates a comprehensive evaluation of how striatal-cerebellar circuit dynamics are modulated across various spontaneous motor contexts, offering novel insights into the amplitude-based signatures of cerebellar compensation [15,16,17]. It is critical to clarify that this mechanical VLS lesion does not intend to replicate the canonical dopaminergic degeneration found in idiopathic Parkinson’s disease [6,7]. Instead, it provides a controlled, circuit-level perturbation of ventral striatal output, allowing us to investigate downstream network destabilization and compensatory processes within the olivocerebellar system that are highly relevant to early-stage Parkinsonian-like dysfunction.

## 2. Materials and Methods

### 2.1. Experimental Subjects and Ethical Compliance

This study was conducted using 24 male Wistar rats (250–350 g; postnatal age 60–80 days), obtained from the Institute of Brain Research at the Universidad Veracruzana. To promote physiological stability and reduce isolation stress, animals were pair-housed in acrylic cages (44 cm × 33 cm × 20 cm) lined with sterile wood shavings. The vivarium environment was maintained under a strictly regulated 12:12 h inverted light–dark cycle (lights off at 07:30 h), ensuring that all experimental manipulations coincided with the subjects’ active circadian phase. Standard rodent chow and water were provided ad libitum. All procedures were performed in strict accordance with the Official Mexican Standard NOM-062-ZOO-1999 [18]. The experimental protocol received formal approval from the Institutional Animal Care and Use Committee: Comité Interno para el Cuidado y Uso de Animales de Laboratorio—CICE (CICUAL-CICE) under code 2018-003, approval date: 1 June 2018.

### 2.2. Study Design and Group Allocation

Subjects were randomly assigned to two independent cohorts: a Control group (*n* = 12), which underwent stereotaxic implantation of recording electrodes in the structures of interest without ventrolateral striatal damage, and an Experimental group (*n* = 12), which received a bilateral VLS lesion in addition to electrode implantation. In both groups, each rat was implanted with its own reference electrode, using the same surgical procedure and stereotaxic coordinates across groups. Group allocation was performed using a computerized sequence generator.

In this study, the Control group did not represent a pre-lesion baseline, but rather a lesion-free physiological reference condition. These animals underwent identical implantation, handling, and recording procedures while preserving intact ventrolateral striatal circuitry, thereby allowing direct comparison of spontaneous motor behaviors and MUA dynamics under normal versus lesion-induced network conditions.

To eliminate observer bias, a single-blind study design was implemented; investigators responsible for behavioral scoring, electrophysiological acquisition, and signal processing were blinded to group assignments until the final statistical analysis. Inclusion criteria were strictly defined to ensure data integrity: only animals exhibiting optimal baseline health (no apparent anomalies), maintained on a standard diet, and completing the full four-week recording regimen without electrode detachment or surgical complications were included.

### 2.3. Characterization of Spontaneous Motor Behaviors

Neural activity was continuously monitored during three distinct spontaneous motor behaviors selected for their ethological relevance: spatial exploration (horizontal and vertical) and self-grooming.

- Horizontal Exploration (Locomotion/Walking): Defined as active ambulatory displacement across the environment, maintaining quaternary limb contact with the floor.
- Vertical Exploration (Rearing): Characterized by the adoption of an orthostatic posture, where the animal supports its weight solely on the hindlimbs to scan the environment [19].
- Self-Grooming (Grooming): This innate maintenance behavior was analyzed according to its syntactic organization, comprising four stereotyped phases: (1) naso-facial grooming, (2) head cleaning, (3) lateral flank grooming, and (4) caudal/anogenital cleaning [9].

Monitoring these specific behaviors enabled assessment of motor circuit integrity in the absence of operant conditioning.

### 2.4. Stereotaxic Surgery and Mechanical Lesioning

Surgical procedures were performed under deep general anesthesia, ketamine (110 mg/kg, Pisa, México City, Mexico); xylazine (8 mg/kg, Cheminova, México City, Mexico). To prevent respiratory obstruction caused by secretions, atropine (0.04 mg/kg; Atropisa Vet, Guadalajara, Mexico) was administered subcutaneously. Subjects were fixed in a high-precision stereotaxic frame (Stoelting Co. 51670, Wood Dale, IL, USA), using Bregma and Lambda as anatomical landmarks for alignment. In the Experimental group, a focal mechanical lesion was induced bilaterally in the VLS (Table 1).

Unlike electrolytic or excitotoxic methods, this mechanical approach, achieved by lowering a stainless-steel electrode (FHC, Inc., Bowdoin, ME, USA, 250 µm *θ*) into the VLS for 30 s without current application, produces a discrete disruption of the striatal microarchitecture. This mimics the focal degeneration often observed in early-stage Parkinsonian dysfunction [15]. Subsequently, monopolar recording electrodes (0.3 MΩ impedance) were chronically implanted into the target cerebellar nodes. Histological verification was performed in all subjects to confirm the sites where the lesion and recording electrodes were placed. The stereotaxic coordinates for all targeted structures are detailed in Table 1.

The implant assembly was anchored to the skull using stainless-steel screws and secured with dental-grade methyl methacrylate to ensure long-term stability.

### 2.5. Electrophysiological Acquisition

MUA signals were acquired using a PolyView 16 system interfaced with Grass Technologies 15A54 amplifiers, Warwick, RI, USA. The raw extracellular signal was amplified (×10,000 gain) and digitally filtered with a band-pass of 100–6000 Hz, and offline digital notch filters at 60 Hz and its harmonics were applied to minimize residual line noise without distorting spike-related transients or the distribution of MUA amplitudes. This broad frequency range was specifically selected to capture high-frequency somatic spiking events (MUA) [21]. To minimize electromagnetic interference, all recordings were conducted within a grounded Faraday cage (150 cm × 150 cm × 50 cm), and a low-torque swivel system (Plastic One Inc., Roanoke, VA, USA) was utilized to allow unrestricted movement during behavioral tasks.

Regarding the weekly recordings, previously implanted animals underwent postoperative treatment with analgesics and antibiotics for five consecutive days, considering Day 1 as the day of surgery. Drug administration was performed every 24 h until completion of the fifth treatment day.

To allow adequate postoperative recovery and minimize pharmacological interference with electrophysiological recordings, the first recording session was conducted on post-surgical day 7. Subsequent recordings were performed on post-surgical days 14, 21, and 28, completing a four-week follow-up period.

Accordingly, Week 1 (W1) corresponded to post-surgical day 7, Week 2 (W2) to day 14, Week 3 (W3) to day 21, and Week 4 (W4) to day 28.

After the final recording session, animals underwent intracardiac perfusion and tissue collection. Brain tissue was fixed in 4% paraformaldehyde for subsequent histological processing, allowing verification of the ventrolateral striatal lesion and accurate electrode placement.

### 2.6. Signal Processing and Amplitude Analysis

Post-acquisition processing involved extracting 30 discrete 5 s traces per animal for each weekly session (10 traces per behavior). To manage the computational load of high-density recordings (50,000 data points per trace) without compromising the statistical representation of the signal distribution, a systematic down-sampling approach was applied. Using the sample size estimation formula for finite populations (95% confidence interval), an optimized subset of 657 data points was extracted from each trace [22].

In the present study, we focused on amplitude-based features of the MUA signal as an indirect index of local population excitability and network gain. This choice reflects the fact that extracellular multiunit amplitudes primarily capture the magnitude of spike-related transients from local neuronal ensembles and can reliably track changes in population activity during movement and other behavioral states [17,21]. While this approach does not resolve single-unit firing statistics or precise temporal coordination, it provides a robust, aggregate measure that is well suited to detect structure- and time-dependent shifts in macroscopic excitability under the present experimental conditions [15,17,21].

The analysis focused exclusively on the amplitude dynamics of the MUA signal. Specifically, the median values of the maximum positive peaks and minimum negative excursions were quantified for each behavioral epoch. The inclusion of both maxima and minima allowed representation of the full amplitude spectrum of neuronal activity, capturing fluctuations in firing-related signal deflections regardless of polarity and preventing bias toward unidirectional amplitude changes. The use of the media provided a robust measure of central tendency, minimizing the influence of sporadic high-amplitude artifacts commonly observed in chronic extracellular recordings.

### 2.7. Histological Verification

Upon completion of the experimental protocols, the subjects were euthanized via systemic administration of sodium pentobarbital (170 mg/kg, i.p.; Cheminova, México City, Mexico) and subsequently perfused transcardially with 0.9% saline followed by 4% paraformaldehyde. Once extracted and post-fixed, the brains were sectioned coronally (45 μm) at −24 °C using a cryostat (Leica Microsystems CM1850, Nussloch, Germany).

To facilitate the visualization of the VLS lesion tracts and cerebellar electrode positioning, the resulting sections were slide-mounted and subjected to a modified cresyl violet (Nissl) stain. Briefly, the tissue was hydrated in distilled water (1 min), incubated in 0.5% cresyl violet acetate (Sigma-Aldrich, St. Louis, MO, USA; C5042-10G) for 8 min, and dehydrated through graded ethanol concentrations (70% for 15 s; 100% for 10 s). Following xylene clearing and mounting with Permount (Fisher Chemical, Waltham, MA, USA), the slides were examined under a light microscope (Olympus Corporation AX70, Tokyo, Japan), which allowed precise quantification of the lesion area and confirmation of anatomical targets.

### 2.8. Statistical Evaluation

Given the non-Gaussian distribution of the electrophysiological amplitude data, as confirmed by Lilliefors and Levene tests, non-parametric statistical approaches were employed.

- Intergroup Analysis: Differences in MUA amplitude between control and experimental groups were assessed at each time point (W1–W4) using the Mann–Whitney U test.
- Intragroup Dynamics: Longitudinal changes within the experimental group were evaluated using the Friedman test. Significant main effects were further explored using post hoc Wilcoxon signed-rank tests with a Bonferroni correction for multiple comparisons.

All statistical analyses were performed in Python 3 using the SciPy and scikit-posthocs libraries, with a significance threshold of *α* = 0.05.

## 3. Results

This study analyzed the amplitude dynamics of MUA across three cerebellar structures: Crus II, the DN, and the IO. The analysis focused on maximum voltage values (positive peaks) and minimum values (negative deflections) during Grooming, Locomotion, and Rearing behaviors across the four recording weeks (W1–W4). Comprehensive mean values are detailed in Appendix A Table A1, Table A2 and Table A3, while the complete statistical analysis is provided in Table A4.

### 3.1. Intergroup Comparisons

#### 3.1.1. Crus II

**Figure 1:**
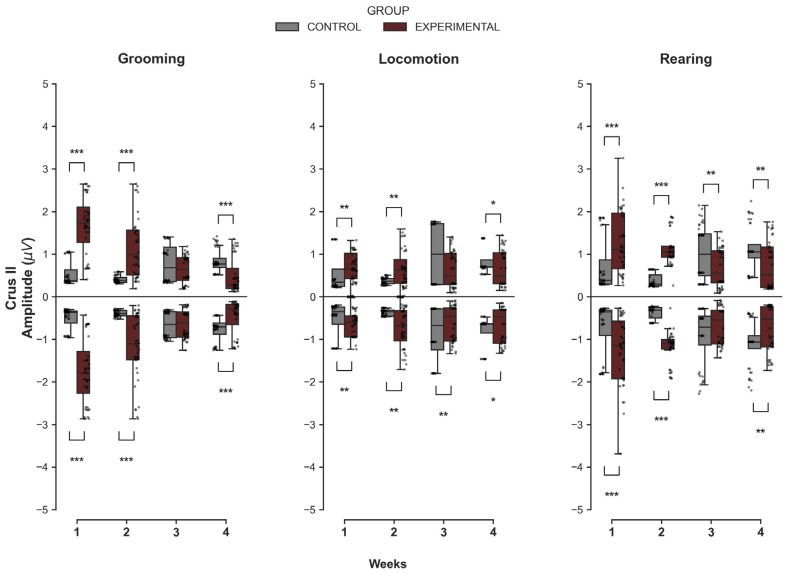
Intergroup comparison of MUA amplitudes over the weeks for Crus II. This box-and-whisker plot shows the median and interquartile range of MUA amplitude (Maximum and Minimum) for the Grooming, Locomotion, and Rearing behaviors in both groups. Individual grey dots represent each recorded amplitude value. The horizontal axis represents the week of recording (W1–W4), and the vertical axis shows the voltage values (µV). The Control group is represented in gray, and the Experimental group in wine. Asterisks indicate statistically significant differences, with the following thresholds: *p* < 0.05: *; *p* < 0.01: **; *p* < 0.001: ***.

The comparison between groups in Crus II revealed significant week-dependent differences in MUA magnitude, indicating a distinct transition from initial hyperexcitability to a late-stage attenuation of neuronal excitability (Figure 1). For Grooming, the Experimental group exhibited a distinct activity pattern. During the early phase (W1 and W2), the Experimental group showed significantly higher maximum amplitudes compared to the Control group (U = 107.0, *p* < 0.001 and U = 211.0, *p* < 0.001, respectively), as well as significantly deeper minimum values (U = 1496.0, *p* < 0.001; U = 1307.0, *p* < 0.001). However, by W4, this relationship had undergone an inversion, with the Experimental group showing significantly lower maximum amplitudes (U = 1268.0, *p* < 0.001) and less pronounced minimum values (U = 334.0, *p* < 0.001) than controls. No significant differences were found in W3 (*p* > 0.05).

A similar transition was detected during Locomotion. The Experimental group presented significantly higher discharge peaks in W1 (U = 503.0, *p* = 0.004) and W2 (U = 527.0, *p* = 0.008). Nevertheless, by W4, the Experimental group showed significantly lower maximum amplitudes compared to controls (U = 1040.0, *p* = 0.020). Regarding minimum values, significant differences were observed across all four weeks (*p* < 0.05), with deeper troughs initially, followed by a reduction in negative amplitude towards the end of the protocol.

Finally, for Rearing, the Experimental group showed greater maximum amplitude in W1 (U = 364.0, *p* < 0.001) and W2 (U = 28.0, *p* < 0.001). Consistent with other behaviors, a transition was observed starting in W3, in which the Experimental group exhibited significantly lower MUA amplitudes than the Control group (W3: U = 1090.0, *p* = 0.005; W4: U = 1118.0, *p* = 0.002).

#### 3.1.2. Dentate Nucleus

**Figure 2:**
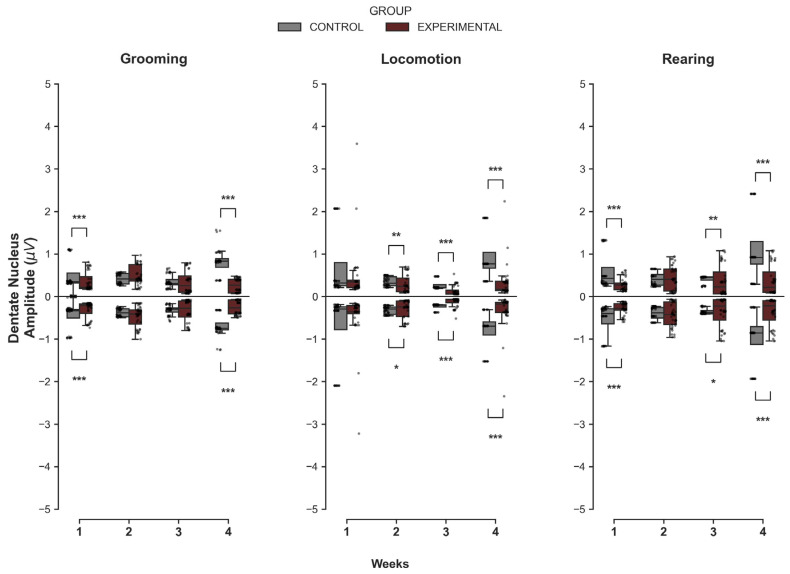
Intergroup comparison of MUA amplitudes over the weeks for the DN. This box-and-whisker plot shows the median and interquartile range of MUA amplitude for behaviors in both groups. Individual grey dots represent each recorded amplitude value. The horizontal axis represents the recording week (W1–W4), and the vertical axis shows the voltage (µV). The Control group is represented in gray, and the Experimental group in wine. Asterisks indicate statistically significant differences, with the following thresholds: *p* < 0.05 (*), *p* < 0.01 (**), *p* < 0.001 (***).

In contrast to the transition observed in the cerebellar cortex, comparisons in the DN revealed a consistent pattern of reduced signal amplitude in the Experimental group compared to Controls across multiple time points (Figure 2). For Grooming, significant differences were identified at the beginning and end of the protocol. In W1, the Experimental group exhibited significantly lower maximum amplitudes (U = 1176.0, *p* < 0.001) and less pronounced minimum values (U = 372.0, *p* < 0.001) compared to the Control group. This difference re-emerged strongly in W4, where the Experimental group again showed inferior maximum activity (U = 1428.0, *p* < 0.001) and reduced minimum troughs (U = 200.0, *p* < 0.001).

During Locomotion, a sustained difference was observed from the second week onwards. The Experimental group displayed significantly lower maximum peaks in W2 (U = 1084.0, *p* = 0.006), W3 (U = 1290.0, *p* < 0.001), and W4 (U = 1450.0, *p* < 0.001). Similarly, minimum values were significantly less negative (indicating lower signal excursion) in the Experimental group for the same period (*p* < 0.05). Regarding Rearing, the Experimental group showed significantly lower signal amplitude than Controls at most time points. Lower maximum amplitudes were observed in W1 (U = 1252.0, *p* < 0.001), W3 (U = 1070.0, *p* = 0.009), and W4 (U = 1320.0, *p* < 0.001), with concomitant reductions in minimum values (*p* < 0.05).

#### 3.1.3. Inferior Olive

**Figure 3:**
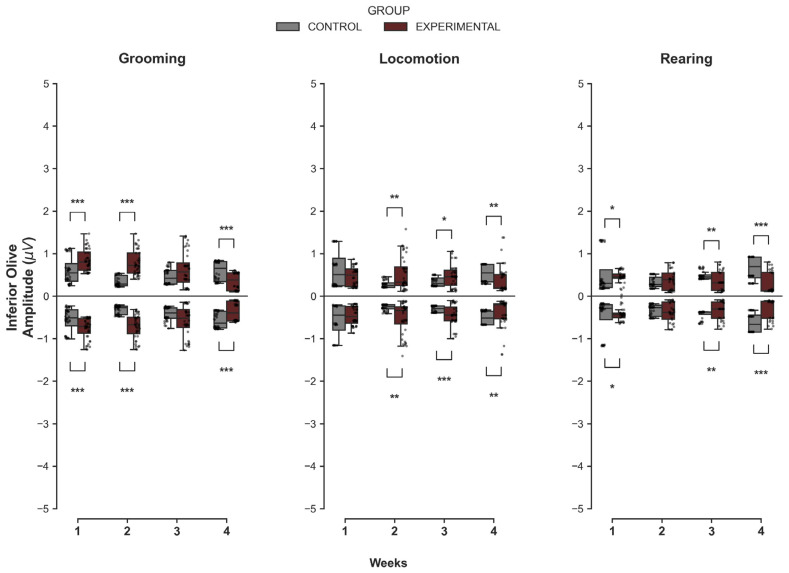
Intergroup comparison of MUA amplitudes over the weeks for the IO. This box-and-whisker plot shows the median and interquartile range of MUA amplitude for behaviors in both groups. Individual grey dots represent each recorded amplitude value. The horizontal axis represents the week of recording (W1–W4), and the vertical axis shows the voltage values (µV). The Control group is represented in gray, and the Experimental group in wine. Asterisks indicate statistically significant differences, with the following thresholds: *p* < 0.05 (*), *p* < 0.01 (**), *p* < 0.001 (***).

Mirroring the trends observed in the cerebellar cortex (Crus II), the IO exhibited a pronounced transition from early-stage hyperexcitability to late-stage attenuation within the Experimental cohort (Figure 3). During Grooming, the Experimental group initially exhibited signs of hyperactivity. In W1 and W2, this group showed significantly higher maximum amplitudes (U = 418.0, *p* < 0.001; U = 96.0, *p* < 0.001) than Controls. However, by W4, the pattern reversed completely: the Experimental group displayed significantly lower maximum amplitudes (U = 1200.0, *p* < 0.001) and less pronounced minimum values (U = 400.0, *p* < 0.001).

For Locomotion, the Experimental group maintained higher activity levels for a longer duration, with significant increases in amplitude observed in W2 and W3 (*p* < 0.05). Nevertheless, by W4, the transition to hypoactivity occurred, as evidenced by significantly lower maximum amplitudes (U = 1140.0, *p* = 0.001). In the case of Rearing, the transition to hypoactivity occurred earlier; while the Experimental group showed higher maximum amplitudes in W1 (*p* = 0.041), by W3 and W4, it exhibited significantly lower amplitudes than Controls (*p* < 0.01).

### 3.2. Intragroup Comparisons

To evaluate whether the differences described above were driven by progressive signal evolution within the lesioned animals, we analyzed the temporal dynamics of MUA exclusively within the Experimental group across the four weeks (Supplementary Materials). Overall, the intragroup analysis indicated a stable amplitude profile. The Friedman test revealed no statistically significant changes in the distribution of maximum or minimum MUA values across weeks for Crus II or the DN during Grooming, Locomotion, or Rearing (*p* > 0.05; see Supplementary Materials, Tables S1–S4 and Figures S1 and S2).

In the IO, a statistically significant variation was detected for Grooming (Friedman test, *p* < 0.05). However, subsequent *post-hoc* pairwise comparisons (Wilcoxon test with Bonferroni’s correction) yielded no significant differences for specific week pairs (Supplementary Materials, Tables S5 and S6). No significant temporal changes were detected for the IO during Locomotion or Rearing. These results suggest that the intergroup differences reported in the previous sections are primarily driven by sustained disparities in activity levels relative to the healthy condition, rather than by a significant progressive drift in signal amplitude within the Experimental group itself (Supplementary Materials, Figure S3).

### 3.3. Corroboration of Lesion Site

**Figure 4:**
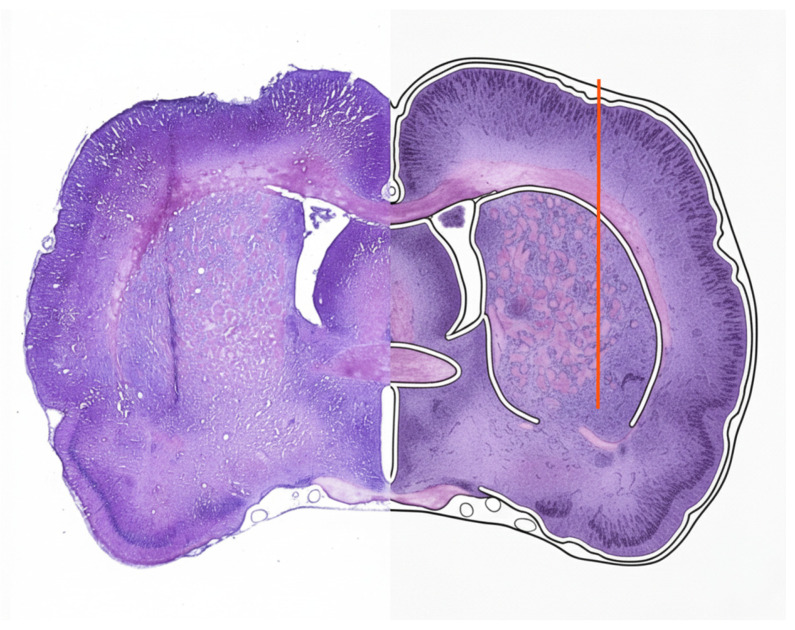
Histological validation of the ventrolateral striatal lesion site. This composite image illustrates the precision of experimental intervention. (Left) Micrograph of a representative coronal section stained with Nissl showing the focal track of the lesion within the VLS. (Right) A hemispheric schematic overlaid on the histological section, where the vertical red line indicates the target lesion depth, serving as a spatial reference to confirm its proper extension according to stereotaxic landmarks. This side-by-side comparison facilitates the direct correlation between the actual tissue disruption and the intended anatomical coordinates.

Upon the conclusion of the four-week recording protocol, the anatomical localization and spatial extent of the mechanical perturbation were meticulously verified in all experimental subjects. Only animals in which the histological assessment confirmed that the focal lesion accurately reached the ventrolateral striatum were retained for final analysis, as verified by post-mortem examination of coronal sections stained with Nissl at low magnification (4×; Figure 4). This rigorous corroboration ensured that all electrophysiological and behavioral results reported herein corresponded to a well-defined, correctly localized, and functionally relevant VLS lesion, thereby excluding any subjects with off-target electrode trajectories or insufficient tissue disruption.

## 4. Discussion

Our findings indicate that a focal mechanical injury to the VLS does not merely produce a local deficit within the basal ganglia circuits; rather, it triggers a widespread, distributed reorganization across the striato-cerebellar network. This is evidenced by the structure-specific, time-dependent modulation of MUA amplitude in Crus II, the IO, and the DN during spontaneous motor behaviors. In parallel, the preservation of the syntactic organization of grooming and exploratory patterns suggests that this reorganization is functionally effective, allowing the maintenance of ethologically relevant behaviors despite persistent circuit perturbation. This adaptive profile is consistent with the notion that cerebellar nodes can assume compensatory roles when basal ganglia output becomes unstable, as reported in neuroimaging studies of Parkinson’s disease, which report cerebellar hyperactivation associated with reduced striatal activity during motor tasks [17,23].

This functional remodeling is supported by the extensive subcortical pathways that indirectly link the basal ganglia to the cerebellum. Specifically, projections from the internal globus pallidus and subthalamic nucleus to the pontine nuclei serve as a major source of excitatory mossy fiber input to the cerebellar cortex and deep nuclei, providing a direct substrate for striatal dysfunction to alter cerebellar dynamics [3,15].

A lesion of the VLS primarily compromises behaviors dependent on prior learning or action value, because the ventral striatum, including the nucleus accumbens, integrates projections from limbic structures such as the amygdala and hippocampus to encode goal-directed movement and performance value [24]. While the dorsal striatum is critical for the execution of habitual actions, ventral striatal damage is known to impair the expression of conditioned responses, particularly those mediated by subregions involved in stimulus-outcome associations [25,26,27].

Furthermore, experimental work has shown that regional striatal lesions can differentially affect the motivational, executive, and motor components of behavior, with ventral striatal lesions exerting a prominent influence on effort allocation and reward-related performance [28]. In this context, a marked disruption of learned behaviors might be anticipated; however, our results reveal that the gross kinematic patterns of horizontal and vertical exploration are behaviorally preserved. This paradox, maintained behavior amidst electrophysiological reorganization, suggests that spontaneous exploration does not rely solely on striatal integrity but rather on the functional state of a broader subcortical network that integrates execution, context, and valuation signals.

Spatial exploration is critical for environmental mapping and requires continuous integration of postural control, locomotion, and three-dimensional coordination [8]. Consequently, while the VLS lesion may not abolish exploration per se, it likely destabilizes the underlying network state, compelling the cerebellum to assume a compensatory role to preserve functionally adequate exploratory patterns. The analysis of self-grooming reinforces this interpretation. This innate behavior, organized into complex syntactic chains of stereotyped movements [9], depends critically on the striatum for temporal sequencing [10]. The fact that grooming remains phenotypically intact while Crus II and IO activity is profoundly altered implies that the cerebellum actively participates in the temporal stabilization of motor sequences when striatal output becomes “noisy” or imprecise.

Striatal spiny projection neurons normally encode key kinematic parameters, such as body velocity and turning speed [11,12]. At the micro-circuit level, this function emerges from the interplay between direct (dSPN) and indirect (iSPN) pathway neurons, which are subject to distinct dopaminergic plasticity rules [29,30]. A VLS lesion likely degrades the signal-to-noise ratio of this output. We propose that the cerebellar responses observed herein are a direct consequence of this striatal destabilization. In this sense, our use of the term “compensation” refers to functional sufficiency rather than complete behavioral normalization: the preserved syntactic organization of grooming and the maintenance of gross exploratory patterns indicate that the cerebellar reorganization is sufficient to sustain ethologically relevant motor sequences, while more subtle kinematic or timing abnormalities may still be present but remain undetected with the present behavioral resolution.

The dynamic transition observed in Crus II, characterized by an early phase of MUA amplitude hyperexcitability followed by late-stage attenuation, suggests a biphasic adaptive process. Early hyperactivity likely reflects an acute compensatory increase in internal gain to counteract unstable striatal input, a phenomenon consistent with the cerebellum’s role in rapid error correction [17,31]. However, the subsequent reduction in activity at W4 indicates a shift towards homeostatic downregulation. This transition may represent a protective mechanism that prevents excitotoxicity or metabolic exhaustion, aligning with models of cerebellar plasticity in which prolonged high-gain states are eventually scaled down to a new, more efficient equilibrium [13,32,33].

Importantly, intergroup comparisons reveal this transition as a reversal in the relationship between lesioned and control animals over time, whereas intragroup analyses show that MUA amplitude within the Experimental group remains relatively stable across weeks. This pattern is compatible with a rapid shift in the lesioned network into a new excitability regime, after which cerebellar output fluctuates around a modified but stable set point, while the control network continues to evolve along a different trajectory.

Within this framework, we use the term “homeostatic regulation” to denote a network-level adjustment of gain rather than a specific cellular or molecular mechanism. Cerebellar nodes initially increase their effective excitability, reflected here in higher amplitudes relative to controls, to counteract the loss of striatal fidelity, and subsequently reduce this gain once motor performance is stabilized. This interpretation is in line with conceptual models in which cerebellar circuits flexibly modulate their contribution to motor control and adaptation when basal ganglia loops are compromised [5,15,23,34].

In contrast, the DN exhibited a distinct profile characterized by sustained hypoactivity or stability, rather than dynamic transition. As the final integrator of cerebellar output, the DN appears to function as a “stabilizing filter,” dampening excessive cortical fluctuations before they propagate to thalamocortical circuits [15]. Anatomical and physiological studies indicate that deep cerebellar nuclei integrate convergent cortical and olivary inputs and relay a temporally shaped output to motor and premotor thalamic targets, thereby exerting a strong influence on the stability of downstream motor commands [33]. This differential response highlights the hierarchical nature of cerebellar processing: while the cortex (Crus II) and olive engage in dynamic gain modulation, the deep nuclei maintain a clamped output to preserve basic motor execution. Our data, showing consistently reduced DN amplitudes in lesioned animals compared to controls, are consistent with DN serving as a bottleneck that limits excessive variability generated upstream, helping to stabilize motor output despite high-gain compensatory signals in Crus II and the IO [33].

The IO mirrored the cortical transition, showing marked temporal sensitivity, particularly during self-grooming. Given the IO’s canonical role in generating error signals via climbing fibers [35], this pattern suggests a recalibration of temporal prediction mechanisms. The early olivary overactivity may reflect the detection of significant timing errors in the grooming sequence immediately post-lesion, which subsides as the network successfully reorganizes [13,31]. It is also plausible that non-synaptic mechanisms, such as local glial activation or tissue remodeling at the lesion site, contribute to the stabilization of these signals over the four-week period [36]. Furthermore, theoretical and computational work has emphasized that plasticity at the IO–deep nuclei synapses can accelerate convergence of cerebellar learning, providing a potential substrate for the rapid reconfiguration observed in our acute lesion model [35].

Collectively, these findings support a model in which VLS injury induces functional instability in the subcortical motor network. The cerebellum responds not as a passive recipient, but as an adaptive controller: Crus II and the IO undergo a dynamic gain recalibration (hyper- to hypo-excitability) to compensate for striatal noise, while the DN maintains a stable output floor. This coordinated reorganization allows for the successful preservation of spontaneous motor behaviors despite significant upstream dysfunction.

Although our mechanical VLS lesion does not reproduce dopaminergic degeneration, the pattern of cerebellar engagement we observe resonates with compensatory cerebellar recruitment described in Parkinson’s disease. Functional MRI and PET studies have reported increased cerebellar activation in patients with PD during motor tasks, often interpreted as a compensatory response to reduced basal ganglia function [13,33,34]. At the same time, other work suggests that cerebellar involvement can also contribute to tremor and other abnormal motor patterns [17,37]. Our results provide an experimental analogue of these observations by showing that, even in an acute ventrostriatal lesion model, cerebellar circuits can rapidly adjust their gain to maintain the integrity of complex spontaneous behaviors, while reshaping the distribution of activity across cerebellar nodes.

### Limitations

First, the use of MUA recordings precludes the identification of specific neuronal sub-types; however, this aggregate signal provides a robust readout of macroscopic network excitability that correlates strongly with regional metabolic demand and constitutes a reliable indicator of global network activity. In addition, the present study stems from a lesion-induced tremor model whose primary objective was to characterize the neuronal activity associated with the presence of mandibular tremors. For this reason, the behavioral analysis was not oriented toward the detailed quantification of kinematic parameters, such as movement velocity or execution precision. Our behavioral assessment was therefore focused on confirming the execution of grooming, locomotion, and rearing patterns, all of which were consistently observed across experimental subjects, without being quantified beyond their relationship with regional neuronal activation patterns.

Similarly, although our behavioral analysis focused on the temporal architecture of spontaneous sequences rather than fine-scale kinematics, the preservation of the syntactic structure in grooming and exploration suggests that the observed cerebellar reorganization was sufficient to maintain functional efficacy, effectively masking potential micro-kinematic deficits that would otherwise require specialized high-speed motion capture systems for detection. Furthermore, the mechanical lesion of the VLS serves as an acute model that isolates immediate plasticity triggers, offering a unique window into the early compensatory “shock response” that is often obscured in chronic neurodegenerative models.

Finally, although the recording montage was restricted to Crus II, the IO, and the DN, these findings establish a critical baseline for the olivocerebellar loop, warranting future investigations to map the full spatial extent of adaptation within this distributed network. Accordingly, our mechanistic interpretation in terms of “gain control” is grounded in amplitude-based population measures and should not be taken as a direct statement regarding changes in single-unit firing statistics, synchrony, or specific oscillatory regimes.

## 5. Conclusions

The present study provides compelling evidence that a focal mechanical lesion of the VLS does not operate in isolation; rather, it triggers a rapid, distributed reorganization of the striato-cerebellar network. Our electrophysiological data reveal a structure-specific compensatory mechanism: while the cerebellar cortex (Crus II) and the IO engage in a “dynamic transition,” shifting from early-stage hyperexcitability to late-stage attenuation, the DN maintains a stable, dampened output profile. Crucially, this neurophysiological remodeling occurs in the absence of overt behavioral deficits. The preservation of the syntactic architecture of grooming and exploration suggests that these cerebellar adjustments effectively counterbalance the loss of striatal fidelity, maintaining motor performance through active homeostatic regulation. These findings challenge the traditional view of the cerebellum as a passive bystander in basal ganglia disorders, positioning it instead as a critical adaptive node that modulates network gain to mask early Parkinsonian dysfunction.

## Figures and Tables

**Table 1:** Stereotaxic coordinates relative to Bregma and functional relevance. AP = Anteroposterior; ML = Mediolateral; DV = Dorsoventral. Coordinates derived from Paxinos and Watson [20].

Structure	AP [mm]	ML [mm]	DV [mm]
VLS	−0.48	±4.40	−6.80
Crus II	−14.00	3.40	−5.00
OI	−11.80	0.80	−11.00
DN	−11.30	3.40	−6.40

## Data Availability

The text fully presents all results from the statistical tests, including median values. Readers requiring access to the underlying dataset for further analysis or verification may contact the corresponding authors.

## Supplementary Materials

The following supporting information can be downloaded at: https://www.mdpi.com/article/10.3390/medsci14010083/s1, Figure S1: MUA amplitude over the weeks in the Experimental group for Crus II; Figure S2: MUA amplitude over the weeks in the Experimental group for the DN; Figure S3: MUA amplitude over the weeks in the Experimental group for the IO; Table S1: Statistical values for Crus II; Table S2: *p*-Values for Crus II; Table S3: Statistical values for DN; Table S4: *p*-Values for DN; Table S5: Statistical values for IO; Table S6: *p*-Values for IO.

## Abbreviations

The following abbreviations are used in this manuscript:

AP	Anteroposterior
DN	Dentate Nucleus
dSPN	Direct pathway spiny projection neurons
DV	Dorsoventral
IO	Inferior Olive
IQR	Interquartile range
iSPN	Indirect pathway spiny projection neurons
ML	Mediolateral
MUA	Multi-Unit Activity
SPN	Spiny projection neurons
TJM	Tremulous jaw movements
VLS	Ventrolateral striatum

## Appendix A

### Appendix A.1. Median Values of Amplitude (Maximum and Minimum)

This section presents the key values for each analyzed structure. Table A1, Table A2 and Table A3 display the normalized amplitude values corresponding to the electrical activity recorded across the target structures during the evaluated behaviors.

**Table A1:** Amplitude values for Crus II. This table presents the calculated median values and interquartile ranges (IQR) for Maximum and Minimum amplitudes across the weeks and behaviors in both groups for Crus II.

Structure	Behavior	Measure	Week	Control [µV]			Experimental [µV]		
				Median	Q1	Q3	Median	Q1	Q3
Crus II	Grooming	Maximum	1	0.379	0.359	0.635	1.729	1.28	2.108
			2	0.389	0.338	0.453	0.985	0.512	1.565
			3	0.687	0.357	1.165	0.637	0.391	0.92
			4	0.771	0.692	0.902	0.281	0.193	0.667
		Minimum	1	−0.368	−0.616	−0.337	−1.787	−2.264	−1.285
			2	−0.393	−0.448	−0.319	−1.104	−1.477	−0.444
			3	−0.649	−0.961	−0.35	−0.616	−0.948	−0.355
			4	−0.707	−0.877	−0.616	−0.278	−0.651	−0.178
	Locomotion	Maximum	1	0.343	0.244	0.651	0.654	0.429	1.022
			2	0.364	0.309	0.428	0.623	0.32	0.871
			3	0.999	0.29	1.719	0.489	0.288	1.018
			4	0.703	0.695	0.87	0.487	0.307	1.036
		Minimum	1	−0.328	−0.643	−0.218	−0.54	−0.925	−0.394
			2	−0.331	−0.413	−0.291	−0.589	−0.835	−0.288
			3	−0.908	−1.541	−0.279	−0.463	−1.025	−0.264
			4	−0.658	−0.816	−0.619	−0.457	−1.028	−0.275
	Rearing	Maximum	1	0.387	0.294	0.868	0.985	0.84	1.127
			2	0.294	0.255	0.512	1.107	0.992	1.341
			3	1.001	0.495	1.477	0.463	0.27	1.106
			4	1.057	0.916	1.225	0.686	0.537	0.845
		Minimum	1	−0.347	−0.793	−0.284	−0.959	−1.139	−0.795
			2	−0.283	−0.492	−0.24	−1.031	−1.3	−0.915
			3	−0.9	−1.332	−0.479	−0.43	−1.007	−0.231
			4	−1.011	−1.161	−0.871	−0.602	−0.782	−0.461

**Table A2:** Amplitude values for DN. This table presents the calculated median values and IQR for Maximum and Minimum amplitudes across the weeks and behaviors in both groups for the DN.

Structure	Behavior	Measure	Week	Control [µV]			Experimental [µV]		
				Median	Q1	Q3	Median	Q1	Q3
Dentate Nucleus	Grooming	Maximum	1	0.479	0.316	0.749	0.198	0.134	0.301
			2	0.404	0.334	0.467	0.444	0.192	0.607
			3	0.334	0.187	0.533	0.438	0.177	0.536
			4	0.224	0.183	0.252	0.548	0.471	0.697
		Minimum	1	−0.446	−0.709	−0.292	−0.193	−0.306	−0.13
			2	−0.38	−0.437	−0.316	−0.428	−0.573	−0.176
			3	−0.309	−0.485	−0.178	−0.413	−0.518	−0.151
			4	−0.21	−0.239	−0.171	−0.528	−0.662	−0.445
	Locomotion	Maximum	1	0.25	0.216	0.366	0.192	0.165	0.222
			2	0.251	0.215	0.276	0.161	0.134	0.193
			3	0.341	0.225	0.436	0.165	0.113	0.201
			4	0.256	0.203	0.324	0.142	0.106	0.171
		Minimum	1	−0.231	−0.354	−0.201	−0.233	−0.279	−0.196
			2	−0.236	−0.259	−0.198	−0.184	−0.223	−0.16
			3	−0.32	−0.423	−0.211	−0.181	−0.228	−0.137
			4	−0.237	−0.306	−0.19	−0.161	−0.194	−0.133
	Rearing	Maximum	1	0.356	0.282	0.429	0.194	0.162	0.227
			2	0.264	0.225	0.301	0.269	0.179	0.355
			3	0.255	0.199	0.287	0.177	0.119	0.248
			4	0.317	0.247	0.407	0.158	0.116	0.208
		Minimum	1	−0.334	−0.406	−0.27	−0.219	−0.258	−0.184
			2	−0.243	−0.285	−0.21	−0.252	−0.316	−0.162
			3	−0.241	−0.271	−0.182	−0.197	−0.276	−0.136
			4	−0.301	−0.385	−0.233	−0.175	−0.225	−0.125

**Table A3:** Amplitude values for IO. This table presents the calculated median values and IQR for Maximum and Minimum amplitudes across the weeks and behaviors in both groups for the IO.

Structure	Behavior	Measure	Week	Control [µV]			Experimental [µV]		
				Median	Q1	Q3	Median	Q1	Q3
Inferior Olive	Grooming	Maximum	1	0.353	0.211	0.54	0.812	0.49	1.134
			2	0.212	0.179	0.313	0.916	0.592	1.341
			3	0.312	0.199	0.413	0.485	0.319	0.692
			4	0.264	0.19	0.353	0.316	0.192	0.444
		Minimum	1	−0.331	−0.518	−0.195	−0.762	−1.047	−0.461
			2	−0.198	−0.285	−0.165	−0.849	−1.258	−0.541
			3	−0.287	−0.394	−0.184	−0.457	−0.662	−0.303
			4	−0.252	−0.32	−0.176	−0.298	−0.413	−0.179
	Locomotion	Maximum	1	0.254	0.203	0.324	0.314	0.19	0.456
			2	0.251	0.215	0.276	0.451	0.334	0.692
			3	0.341	0.225	0.436	0.489	0.319	0.697
			4	0.256	0.203	0.324	0.256	0.183	0.353
		Minimum	1	−0.237	−0.306	−0.19	−0.302	−0.428	−0.177
			2	−0.236	−0.259	−0.198	−0.428	−0.662	−0.306
			3	−0.32	−0.423	−0.211	−0.463	−0.662	−0.303
			4	−0.237	−0.306	−0.19	−0.241	−0.331	−0.171
	Rearing	Maximum	1	0.356	0.282	0.429	0.493	0.316	0.697
			2	0.264	0.225	0.301	0.301	0.19	0.444
			3	0.255	0.199	0.287	0.456	0.319	0.692
			4	0.317	0.247	0.407	0.194	0.162	0.227
		Minimum	1	−0.334	−0.406	−0.27	−0.461	−0.662	−0.301
			2	−0.243	−0.285	−0.21	−0.285	−0.413	−0.177
			3	−0.241	−0.271	−0.182	−0.428	−0.662	−0.301
			4	−0.301	−0.385	−0.233	−0.183	−0.212	−0.151

### Appendix A.2. Intergroup Statistical Analysis

This section presents the comprehensive statistical evaluation of the differences between the Control and Experimental groups across all recording weeks (W1–W4). The Table A4 below details the full output of the Mann–Whitney U tests applied to the MUA amplitude for Crus II, DN, and IO.

**Table A4:** Intergroup statistical comparison of MUA amplitude dynamics. Mann–Whitney U test results comparing MUA amplitude between Control and Experimental groups for Crus II, the DN, and the IO. Statistics represent maximum positive peaks and minimum negative excursions recorded during Grooming, Locomotion, and Rearing across the four-week protocol (W1–W4).

Structure	Behavior	Week	Maximum		Minimum	
			U Statistic	*p*-Value	U Statistic	*p*-Value
Crus II	Grooming	1	107	<0.001	1496	<0.001
		2	211	<0.001	1307	<0.001
		3	957	0.132	754	0.662
		4	1268	<0.001	334	<0.001
	Locomotion	1	503	0.004	1095	0.005
		2	527	0.009	1108	0.003
		3	1000	0.054	470	0.001
		4	1040	0.021	570	0.026
	Rearing	1	364	<0.001	1215	<0.001
		2	28	<0.001	1574	<0.001
		3	1090	0.005	690	0.291
		4	1118	0.002	522	0.007
Dentate Nucleus	Grooming	1	1176	<0.001	372	<0.001
		2	703	0.353	887	0.405
		3	902	0.329	700	0.338
		4	1428	<0.001	200	<0.001
	Locomotion	1	995	0.061	810	0.927
		2	1084	0.006	592	0.045
		3	1290	<0.001	310	<0.001
		4	1450	<0.001	230	<0.001
	Rearing	1	1252	<0.001	414	<0.001
		2	814	0.896	820	0.851
		3	1070	0.009	590	0.043
		4	1320	<0.001	320	<0.001
Inferior Olive	Grooming	1	418	<0.001	1158	<0.001
		2	96	<0.001	1486	<0.001
		3	640	0.125	963	0.118
		4	1200	<0.001	400	<0.001
	Locomotion	1	978	0.087	715	0.416
		2	504	0.004	1078	0.007
		3	578	0.033	1142	<0.001
		4	1140	0.001	460	0.001
	Rearing	1	428	0.041	782	0.031
		2	553	0.579	637	0.663
		3	861	0.002	341	0.002
		4	1000	<0.001	200	<0.001
