# Supplementary material for: Changes in Cerebellar Multiunit Activity Associated with Ventrolateral Striatal Injury During Spontaneous Motor Behavior
Source: Med Sci (Basel). 2026 Feb 11;14(1):83. doi: 10.3390/medsci14010083 (PMC12921778; doi:10.3390/medsci14010083)
Supplement: Supplementary file 1 [file medsci-14-00083-s001.zip › medsci-4115887-supplementary.pdf]

Supplementary materials 1

Statistical for Intragroup Comparison

This section summarizes the results of the Friedman and Wilcoxon tests with Bonferroni adjustment for the intragroup analysis conducted exclusively on the Experimental group. The analysis was performed independently for each cerebellar structure: Crus II, Dentate Nucleus (DN), and Inferior Olive (IO). To provide a comprehensive view of signal evolution, we present both tabulated statistical test values and corresponding longitudinal plots (Figures and Tables), illustrating the distribution of MUA amplitude across the four recording weeks.

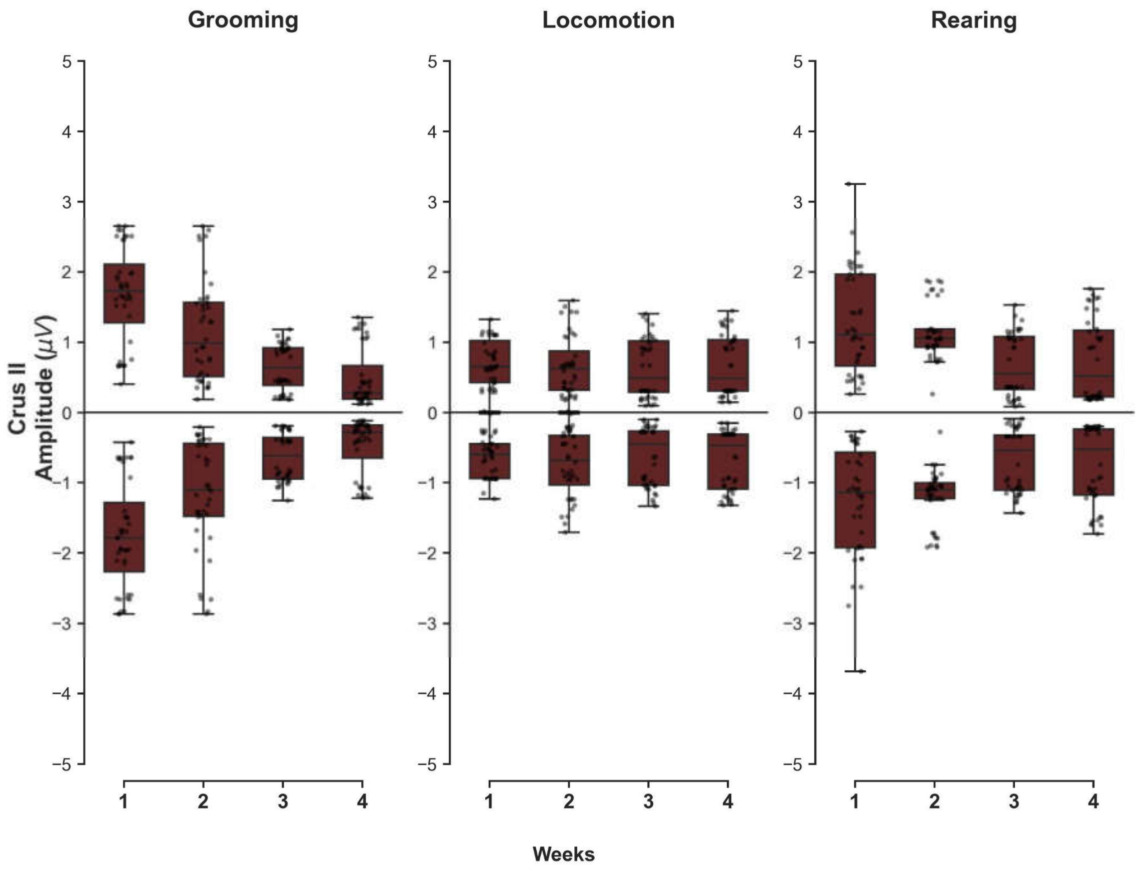

Figure S1. MUA amplitude over the weeks in the Experimental group for Crus II. This box-and-whisker plot displays the median and interquartile range of MUA amplitude values for behaviors in the Experimental group. Individual grey dots represent each recorded amplitude value. The horizontal axis represents the recording weeks (W1–W4), while the vertical axis shows the voltage values (μV).

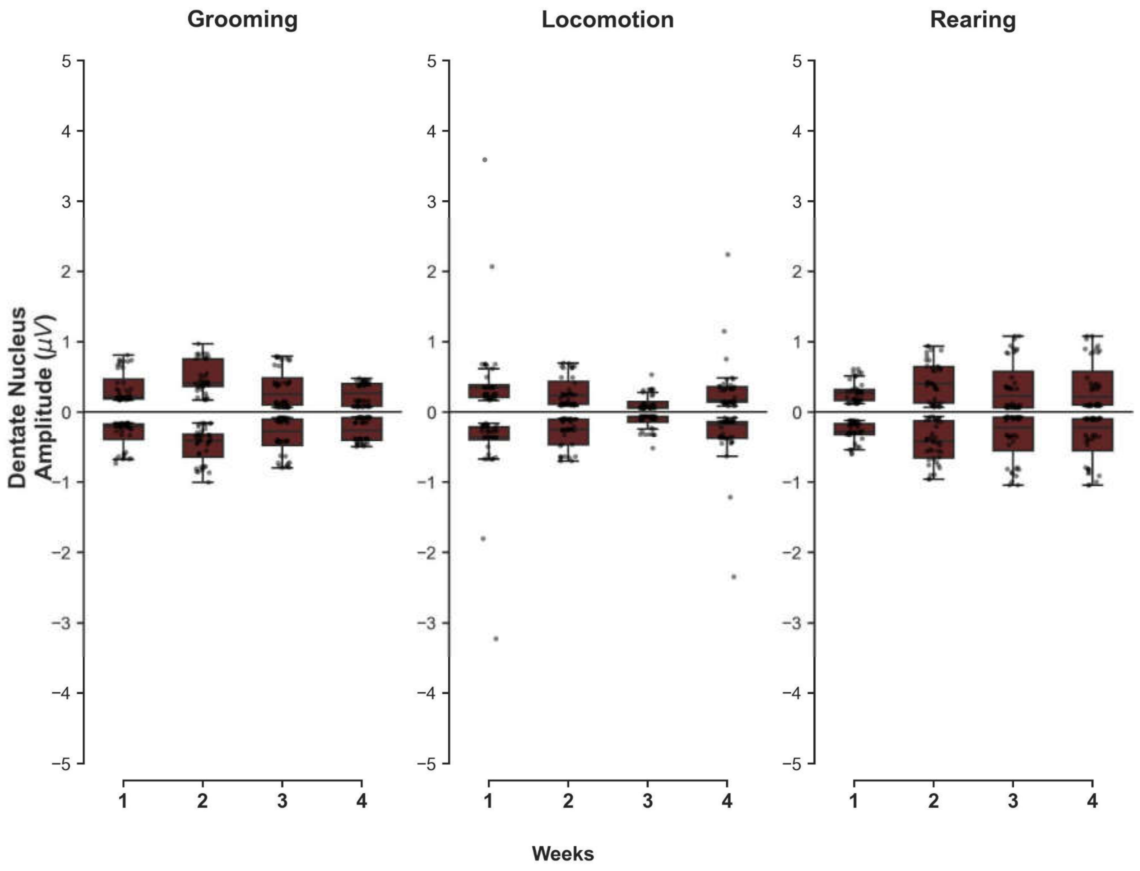

Figure S2. MUA amplitude over the weeks in the Experimental group for the DN. This box-and-whisker plot displays the median and interquartile range of MUA amplitude values for behaviors in the Experimental group. Individual grey dots represent each recorded amplitude value. The horizontal axis represents the recording weeks (W1–W4), while the vertical axis shows the voltage values (μV).

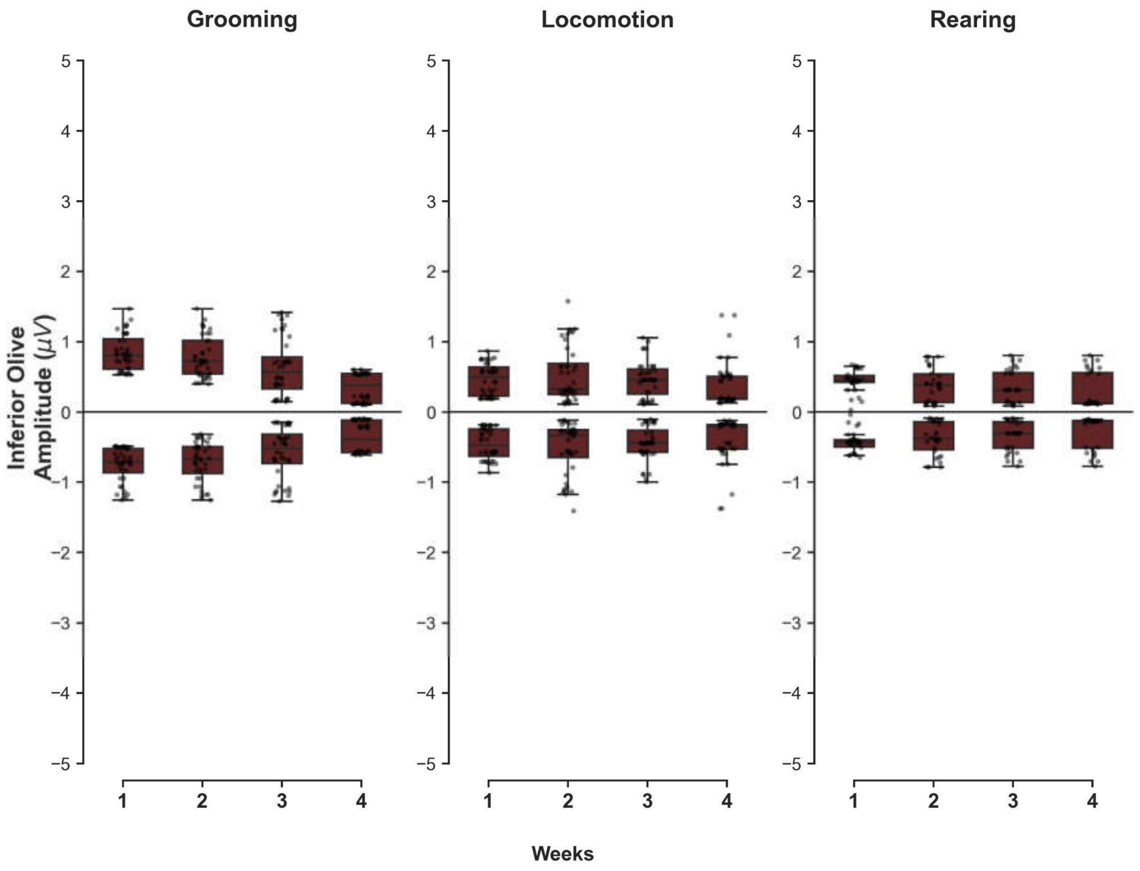

Figure S3. MUA amplitude over the weeks in the Experimental group for the IO. This box-and-whisker plot displays the median and interquartile range of MUA amplitude values for behaviors in the Experimental group. Individual grey dots represent each recorded amplitude value. The horizontal axis represents the recording weeks (W1–W4), while the vertical axis shows the voltage values (μV).

Structure	Behavior	Measure	Friedman Statistical Value	Wilcoxon Statistical by Weeks Compared					
				(1, 2)	(1, 3)	(1, 4)	(2, 3)	(2, 4)	(3, 4)
Crus II	Grooming	Maximum	7.462	0	1	0	1	0	2
		Minimum	7.462	0	1	0	2	0	2
	Locomotion	Maximum	1.421	3	3	5	4	5	1
		Minimum	2.053	2	5	5	3	3	0
	Rearing	Maximum	7.4	1	0	0	0	0	2
		Minimum	7.4	1	0	0	0	0	2

Table S1. Statistical values for Crus II. This table shows the statistical values obtained from the Friedman (Chi-square) and Wilcoxon tests for comparisons between weeks in the Experimental group for Crus II.

Structure	Behavior	Measure	Friedman p-value	Wilcoxon p-value by Weeks Compared					
				(1, 2)	(1, 3)	(1, 4)	(2, 3)	(2, 4)	(3, 4)
Crus II	Grooming	Maximum	0.059	1	1	1	1	1	1
		Minimum	0.059	1	1	1	1	1	1
	Locomotion	Maximum	0.701	1	1	1	1	1	1
		Minimum	0.562	1	1	1	1	1	1
	Rearing	Maximum	0.06	1	1	1	1	1	1
		Minimum	0.06	1	1	1	1	1	1

Table S2. p-Values for Crus II. This table shows the p-values obtained from the Friedman and Wilcoxon tests with Bonferroni adjustment for the comparisons between weeks in the Experimental group for Crus II.

Structure	Behavior	Measure	Friedman Statistical Value	Wilcoxon Statistical by Weeks Compared					
				(1, 2)	(1, 3)	(1, 4)	(2, 3)	(2, 4)	(3, 4)
DN	Grooming	Maximum	4.385	0	5	3	0	1	5
		Minimum	4.385	0	4	3	0	1	4
	Locomotion	Maximum	4.5	1	1	1	2	1	0
		Minimum	4.5	1	1	2	1	1	0
	Rearing	Maximum	1.5	2	5	3	2	5	0
		Minimum	1.5	1	5	3	2	4	0

Table S3. Statistical values for DN. This table shows the statistical values obtained from the Friedman (Chi-square) and Wilcoxon tests for comparisons between weeks in the Experimental group for the DN.

Structure	Behavior	Measure	Friedman Statistical Value	Wilcoxon p-value by Weeks Compared					
				(1, 2)	(1, 3)	(1, 4)	(2, 3)	(2, 4)	(3, 4)
DN	Grooming	Maximum	0.223	1	1	1	1	1	1
		Minimum	0.223	1	1	1	1	1	1
	Locomotion	Maximum	0.212	1	1	1	1	1	1
		Minimum	0.212	1	1	1	1	1	1
	Rearing	Maximum	0.682	1	1	1	1	1	1
		Minimum	0.682	1	1	1	1	1	1

Table S4. p-Values for DN. This table shows the p-values obtained from the Friedman and Wilcoxon tests with Bonferroni adjustment for the comparisons between weeks in the Experimental group for the DN.

Structure	Behavior	Measure	Friedman Statistical Value	Wilcoxon Statistical by Weeks Compared					
				(1, 2)	(1, 3)	(1, 4)	(2, 3)	(2, 4)	(3, 4)
IO	Grooming	Maximum	8.842	0	1	0	1	0	0
		Minimum	11.368	1	0	0	0	0	0
	Locomotion	Maximum	1.923	5	4	1	2	2	3
		Minimum	1.154	5	5	1	2	3	4
	Rearing	Maximum	1.393	2	1	1	0	1	1
		Minimum	1.393	2	1	1	0	1	1

Table S5. Statistical values for IO. This table shows the statistical values obtained from the Friedman (Chi-square) and Wilcoxon tests for comparisons between weeks in the Experimental group for the IO.

Structure	Behavior	Measure	Friedman (p)	Wilcoxon p-value by Weeks Compared					
				(1, 2)	(1, 3)	(1, 4)	(2, 3)	(2, 4)	(3, 4)
IO	Grooming	Maximum	0.031	1	1	1	1	1	1
		Minimum	0.01	1	1	1	1	1	1
	Locomotion	Maximum	0.589	1	1	1	1	1	1
		Minimum	0.764	1	1	1	1	1	1
	Rearing	Maximum	0.707	1	1	1	1	1	1
		Minimum	0.707	1	1	1	1	1	1

Table S6. p-Values for IO. This table shows the p-values obtained from the Friedman and Wilcoxon tests with Bonferroni adjustment for the comparisons between weeks in the Experimental group for the IO.
